# The English of an Elizabethan Physician

**Published:** 1904-10-22

**Authors:** 


### The English of an Elizabethan Physician.

Those who write of the genius of Shakespeare, as shown by his vast knowledge, are a little apt to overlook the marvellous culture of Elizabethan writers in general. Either the school-teaching during the sixteenth century was very much superior to that of the present day, or men's thoughts ran especially along classical lines. Of these alternatives the latter seems the more likely. The English mind has often shown a tendency to run in grooves, thus a hundred years later, in the Stuart times, England was essentially a musical country and Pepys records without any surprise that he met gentlemen casually on the Thames who could sing difficult part songs with him at sight, and he would examine his servants as to their musical attainments and think nothing of it. In our own day conversation on bicycles and motor cars shows a knowledge of machinery which might well have astonished an engineer a few years ago. The following extract gives a good example of the style prevalent during the later years of Elizabeth's reign. It was written by Philip Barrough, a physician possessing a license to practise from the University of Cambridge who wrote a somewhat uninteresting "Method of Physic, containing the causes, signs, and cures of inward diseases in man's body from the head to the foot." In the preface to the reader is the following account of the position of ancient physicians. The tradition about Galen and Hippocrates shows how curiously modern was their attitude towards clinical work. "Many arguments do induce me to believe the witness of historians that Physic is the art wherein many Kings have travailed and delighted: so nothing more strongly than this that whereas other Gods were tied to their several places as Jupiter to Elis, Diana to Ephesus, Apollo to Delos, and so forth, Aesculapius hath his temples and altars everywhere. . . . And I pray how much inferior was the renown of Hippocrates who descended of this line, saving that the one was placed amongst the gods in heaven and the other reverenced as a mortal god upon earth? Did not he make Coos, the place where he was born, of an inglorious island a famous country, only by the access of other nations who were brought thither by the report of his wonderful skill in Physic? What familiarity had he with kings! What estimation among the philosophers of that time as Democrates and others! and to conclude, how was his fame spread universally throughout all Greece! . . . It will easily appear of what credit this noble science of physic was in times past, if you consider the insolency and pride of ancient physicians, whereof many of them disdained the fellowship of Kings and some of them, emboldened only by the credit their science purchased amongst men, grew to such impudency that they would have ceremonies and rites performed unto them as unto gods. One such was Themison Citrius, the dainty of Antiochus, another was Thessalus whom Galen maketh mention of. . . . But the most famous of all was Menecrates, the Syracusan, who foolishly usurped the name of Jupiter, oftentimes boasting that by his art he could breath life into mortal men after the manner of Jupiter: which arrogant title the people never went about to derogate from him, but rather supposed that it was deserved on his part because he cured many of the falling evil (epilepsy) which disease especially reigned in his time. This Menecrates, in a certain epistle which he wrote to Philip, the King of Macedonie, used these words: "Thou art King of Macedon, and I of physic; it lieth in thy power at thy pleasure to destroy men which enjoy their health and in mine to preserve sick men and restore dead men to life, and to keep the health of men unspotted even unto their old age if they will obey me. Unreasonable, surely, and monstrous was the pride of this man, and it was so fed with the applause and approbation of his citizens, who wondered at his rare cunning, that he marched in the city with a train of gods after him: one, in the habit of Hercules, another in the shape of Mercury, another took upon him the form of Apollo, and he himself super-eminent in the midst resembling Jupiter, wore a purple robe and a crown of gold upon his head, and held in his hand a mighty sceptre. The opinion of this science did so possess the minds of the people in those days that they imagined the professor of the same to be sent immediately from heaven, for the commodity of the whole country and for the preservation of mankind: which made them not to doubt to do unto them all superstitious reverence that might be, whereof grew this excessive pride, which hath arrested, as it were, the minds of many physicians. I would not wish that the physicians of our time should draw this unto an example, but rather with all lowliness, to visit even the poorest when their help is required; for, seeing that the life of the most miserable vassal is as dear in the sight of God as the life of the most renowned monarch, shall not the physician look to have a shrewd check at God's hand if he either hath proudly denied his help to the poor, or negligently visited them? I will not descend into this common place, though (to speak the truth) the arrogance of many of our physicians might give me sufficient occasion, wishing them to leave off to imitate the swelling insolency of Menecrates and the rest and to track rather in the steps cf Hippocrates and Galen, of whom it is thus written that they never disdained to shroud themselves under the simplest roof in their country to do the poorest man good: and as they themselves said (besides the glory they purchased by their courteous benignity) they added always to their cunning and by experience confirmed their art and knowledge, which might well stagger, if you respect the infinite variety of diseases and the strange diversity of men's dispositions. And truly, if nothing else, yet the enriching of their knowledge which is gathered especially by long experience, might be cause good enough to attract and draw them to lay hold of any occasion to go to the diseased person. And in mine opinion, the neglecting of this which proceedeth either from covetousness or pride hath been the only cause that even from the beginning there have been reckoned so few good phyicians: for exempt only Hippocrates, Galen, Avicen, Aegineta, Aetius, and Soranus (though the number of common physicians hath been great) you shall not find any that have climbed up to the perfection of their science, nay far from that, which might challenge the fifth or sixth place."

